# Illuminating the Imperceptible, Researching Mina’i Ceramics with Digital Imaging Techniques

**DOI:** 10.3390/jimaging7110233

**Published:** 2021-11-08

**Authors:** Dana Norris, Oliver Watson

**Affiliations:** 1 Cranfield Forensic Institute, Cranfield University, Defence Academy of The United Kingdom, Shrivenham SN6 8LA, UK; 2 The Oriental Institute, University of Oxford, Pusey Lane, Oxford OX1 2LE, UK; oliver.watson@orinst.ox.ac.uk

**Keywords:** Mina’i ceramics, fritware, overglaze enamel, conservation, ultraviolet light, infrared reflectography, radiography

## Abstract

Mina’i ceramics dating to the late 12th and early 13th century made in the Kashan region of Iran represent a novel period of overglaze enamelling technology in ceramic history. New colours were used to produce stylistically attractive and dynamic polychrome motifs. Due to their archaeological context, and popularity in the art market since the mid-20th century, these objects often have complex conditions involving reconstruction and overpainting. The aesthetic and technological significance of these pieces warrants further study, but in practice, removing restorations can lead to structural destabilisation, requiring time-consuming and potentially unplanned for conservation treatment. To determine if it is possible to gain useful information from the study of these artworks without disturbing existing restorations, a group of objects were drawn from the Sarikhani and Ashmolean Museum of Art and Archaeology collections. The objective of this project was twofold, first to assess the merits of the imaging techniques for understanding condition, and second to propose a protocol for imaging with the aim of encouraging collaborative projects with international partners. The techniques used in this study include digital photography under visible and ultraviolet light, infrared reflectography, and radiography. The results show that important information invisible to the naked eye can be obtained about the decorative surfaces, using ultraviolet light and infrared reflectography. Digital radiography proved to be equally effective when studying the condition of the ceramic body. The results of this project were used to produce guidance on these techniques as a collaborative documentation package for the study of Mina’i ceramics.

## 1. Introduction

In the twelfth century, the ceramic landscape of Iran was suddenly and dramatically transformed by the arrival of potters from Egypt, fleeing the political and economic chaos which accompanied the last years of the Fatimid regime. They settled in the town of Kashan, which stood on the edge of the great desert on the main route from the great city of Rayy in the north to the cities of Isfahan, Yazd and Kirman in the centre and south. Kashan was a small town, without a previous history of ceramic manufacture, yet within a few years it became renowned as the place which made and exported the highest quality ceramics across Iran and further afield into Islamic lands. Today, it is recognised as one of the world’s great mediaeval ceramic centres for the quality of its products, its technical and artistic invention, and its trading success. Its heyday did not last long—perhaps 50 years before the Mongol invasions in the early thirteenth century interrupted production for some four decades, and then another 70 years or so of fame under the Mongol Ilkhanid regime. Its fortunes declined along with the Ilkhanid’s in the 1340s.

Yet the decades prior to the Mongol invasions saw an extraordinary burst of invention. The potters brought with them the artificial pure-white “fritware” [1,2] ceramic fabric which for the first time allowed the making of vessels of great fineness, subtlety of profile and brilliant, coloured glazes. They also brought the dazzling lustre-painting technique, which allowed fine decoration with brilliant metallic and mother-of-pearl reflections. The potters developed, for the first time, stable underglaze painting [3], a technique that became the standard for all later fine ceramics. 

However, their most spectacular invention was that of Mina’i decoration—an overglaze enamel technique [3] that allowed for precise painting in brilliant colours. Enhanced at times with gold leaf, it allowed the potters for the first time to produce complex decorative patterns or detailed narrative scenes which could draw on the finest elite arts available—book illustration and wall painting. 

Mina’i wares are of the highest importance in two respects: on the one hand, they represent a towering peak of unforeseen excellence in pottery-making in the mediaeval Islamic world; on the other, they preserve, albeit in small scale, the visual culture of illustrated books and wall-painting from a period in which the originals are only known by a pitiful handful of fragments. Scenes from the great Iranian epic Shahnama, for example, are found on Mina’i wares more than a century earlier than any surviving illustrated manuscript.

### Challenges of Study

However, our understanding of this extraordinary ware is limited. Though it was at first ferociously collected in the 1920s and 1930s, reaching unparalleled sums, it has since rather fallen from interest. It has not inspired anything like the same extensive research as its sibling lustre-ware, made by the same potters at the same time. Few detailed studies have been undertaken: we lack even a basic typology. The bibliography is scanty, reliable information is scarce. Why?

The reasons are quick to see, overglaze enamel decoration is easy to forge and to ‘improve’ [4]. Considerable efforts were taken from the very beginning by unscrupulous art dealers to ‘fancy-up’ plainer pieces with additional painting, to construct new vessels from unrelated sherds, and to make new pieces to fill missing parts. The restorers’ skill means that this work is nigh impossible to spot, though warnings were published from the earliest days. To undertake study without close conservation and technical examination is a very risky business. Curators, academics and collectors have become wary as the extent of fakes, forgeries and overzealous restoration have become more and more apparent [5].

The Sarikhani collection provides an important opportunity to rectify this neglect, and to establish a sound basis for further understanding. The collection contains a significant number of major pieces which, at first sight, seem to be ‘too good to be true’, but on closer inspection, appear in every way to be as excellent as they seem. However, in order to fully understand and appreciate them, detailed work needs to be carried out to strip off later restorations and overpainting, to ensure that we see the original effect. The range of wares in the Sarikhani collection is such that it will provide a solid bedrock of understanding: we will know what the original intention was of the potters and what the original consumers desired. On this basis, we will be able to approach and judge with much more confidence the pieces in other collections which have not undergone such close study. Mina’i can start to take its rightful place in Iranian ceramic history. 

**Figure 1:**
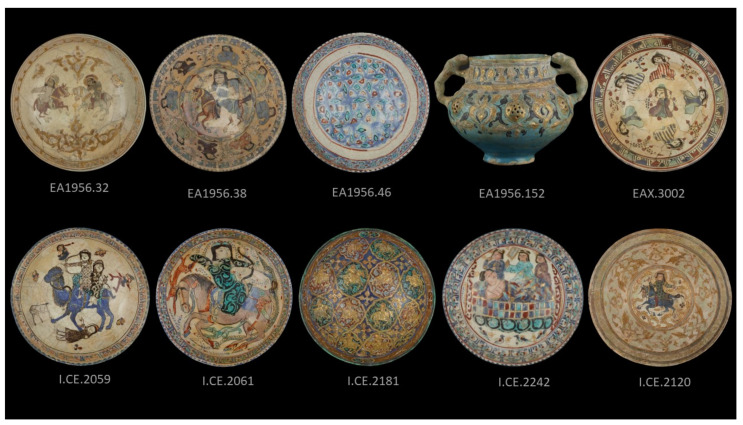
Upper row are objects from the Ashmolean Museum of Art and Archaeology, University of Oxford. Additional views of EA1956.22, EA1956.152, and EAX.3002 are available on the museum website [11,12,13]. The second row are objects from the Sarikhani Collection; see Watson 2020 [10] (pp. 242–280).

This project evaluates the benefits of three imaging techniques established in the study of art and archaeology for their application to the study of Mina’i ceramics. These imaging techniques are digital photography under ultraviolet light (UV) [6,7], infrared reflectography (IR) [8], and radiography (2D X-ray) [6,9]. The equipment and methodology are described in the following sections. The effectiveness of each technique is evaluated in comparison to visual examination in the discussion. The aim of this project was to develop a methodology for the study of Mina’i ceramics which utilizes non-destructive approaches, before moving on to investigative conservation treatments or scientific analysis requiring samples. Recommendations for the study of Mina’i ceramics with digital imaging are presented in the conclusion so that documentation is consistent and directly comparable, in the hope of encouraging collaborative research. Ten objects were included in this study: five from the Sarikhani Collection (I.CE. 2059, I.CE. 2061, I.CE. 2181, I.CE. 2242, and I.CE. 2120) and five from the Ashmolean Museum (EA1956.32, EA1965.38, EA1965.46, EA1956.152 and EAX.3002), which are attributed to the mid-12th to early13th century [10]. See Figure 1.

## 2. Methodology

Before imaging with UV, IR and radiography, each object was examined, and the condition documented with written comments. Existing images of the front and back were annotated to help record the details of examination. Standard digital images were taken under visible light for a reference to the three techniques trialled in this project. Bowls were preferred because the shape is less complex, allowing researchers to focus on the surface decoration, and avoiding multiple walls in the radiographs. The orientation of each view was positioned carefully so it could be replicated during subsequent imaging sessions. High-resolution photographs included a measurement scale and colour card. The initial images were taken in the Ashmolean’s Photography Department by a professional photographer and conservator using a Phase One XF camera with an IQ3 60MP digital back, and Bowens flash lighting. The images were fed directly into a laptop where they could be viewed and the settings were controlled with Capture One software. The RAW images were later processed in Photoshop and saved as JPEGs. A copy stand or tripod was used in combination with a remote shutter release to prevent camera shake, and the images were taken using a black background.

### 2.1. Digital Images under Ultraviolet Light (UV)

UV images were taken just after each of the visible light images, partially to maintain the position of the objects, but also to utilise the expertise of the museum’s professional photographer. UV photography was done in a darkened room, using the same equipment as the standard photography with a black background to mitigate fluorescence from the backdrop. The objects were illuminated and examined under a Panacol hand-held UV lamp model UV-H 255; this lamp produces light at UV-B 280–315 nm and UV-A 315–400 nm frequencies. Notes were taken on the fluorescence for each object because what was visible during examination was much more vibrant than that captured by the camera. For each position, the camera was focused for the standard visible light images, the lights turned off, and the exposure time increased to 30–60 s. The UV lamp was moved around the object to illuminate the surface evenly without moving in front of the lens. Due to the variation in fluorescence, it was necessary to adjust the exposure time for each object to achieve the optimal image. The resulting UV images have a purple hue; they were not saved in greyscale because the colour of the fluorescence given off by the restorations can indicate what type of material it is [14] (p. 36). Safety precautions were taken by wearing protective goggles conforming to British Standard BS EN 170 [15] when operating the UV lamp. Where possible, skin was covered, such as wearing long sleeves and gloves while working for long periods. A notice was posted on the door to the room to prevent people entering the space without protective goggles.

### 2.2. Digital Infrared Reflectography (IR)

IR images were taken with an infrared adapted Lumix Panasonic DMC-GX1 camera fitted with a Polaroid Optics IR720 Filter. The objects were photographed on a grey ground. The artworks were illuminated with two lamps using Philips Photocrescenta 240 V 75 W bulbs; it was not necessary to turn off other lights in the room. The IR images were recorded as RAW files by the camera, transferred to a computer and processed in Photoshop. They were saved as JPEGs in grey scale, removing the pink colour escaping the filter because it is distracting and does not add to the interpretation.

### 2.3. Digital Radiography (2D X-rays)

Radiographs were taken by representatives from SCANNA, a manufacturer of portable X-ray equipment. A XR150 lightweight 270 kV pulsed X-ray generator was used with a digital plate, Panasonic tablet and ScanView x-ray control and imaging software. The exposure time was three pulses. The images were downloaded from the tablet as TIFF files, processed, and saved as JPEGs in Adobe Lightroom CC. In this project, the contrast was set at 20, shadow 20, clarity 50, sharpening 75, and noise reduced by 20. A risk assessment was carried out, and appropriate administrative controls put in place by the Conservation Department and SCANNA Staff. Nearby staff were notified that X-rays were in use and prevented from entering the space during imaging.

## 3. Results

**Figure 2:**
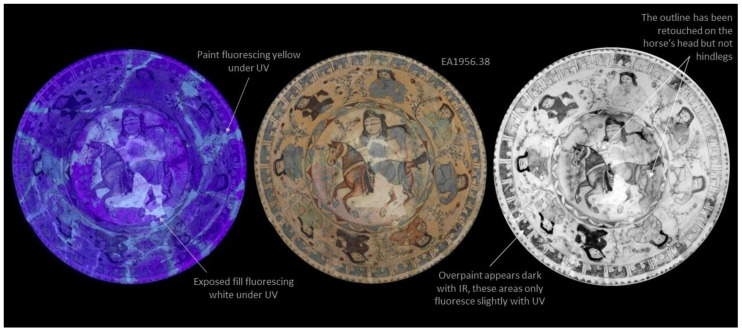
Results of digital photography under ultraviolet light (UV) left, standard visible light centre, and infrared reflectography (IR) on the right for EA.1956.38.

**Figure 3:**
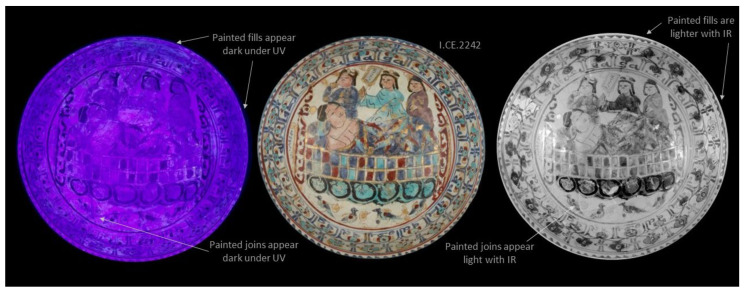
Results of digital photography under ultraviolet light (UV) left, standard visible light centre, and infrared reflectography (IR) on the right for I.CE. 2242.

In Figure 2 and Figure 3, photography under UV light, visible light, and IR images of EA1956.38 and I.CE. 2242 are presented side by side. The standard digital images are positioned centrally to make comparison easier. The UV image of EA1956.38 is typical for a ceramic with some restoration; the ceramic fluoresces purple [9]. The exposed fills which can be easily seen in the standard visible light images fluoresce white, and painted areas fluoresce with a yellow hue; see the annotations in Figure 2. In contrast, on I.CE. 2242 in Figure 3, the painted restorations appear dark in the UV image. These areas are annotated in Figure 3. This result is rare, and these materials may have been selected because they do not fluoresce [6]. Multiple views of EA1956.22, EA1956.152, and EAX.3002 with corresponding UV images are available on the Ashmolean museum website [10,11,12].

Other areas of restoration are highlighted by IR in the images on the right of Figure 2 and Figure 3. Looking at the rim of EA1956.38, some sections of the blue ground appear much lighter than others, and this does not always correspond with the fluorescence in the UV image. This phenomenon is due to the pigments in the paint used as a restoration material absorbing and reflecting infrared light differently than the ceramic. Areas of restoration on the rim of I.CE. 2242 are also more obvious under IR, and these do not fluoresce under UV (at the top, upper right, and upper left). Scrutiny of more obvious areas of restoration helps the researcher understand the unique appearance of the original and applied materials on individual objects, from which point less obvious details can be observed. 

**Figure 4:**
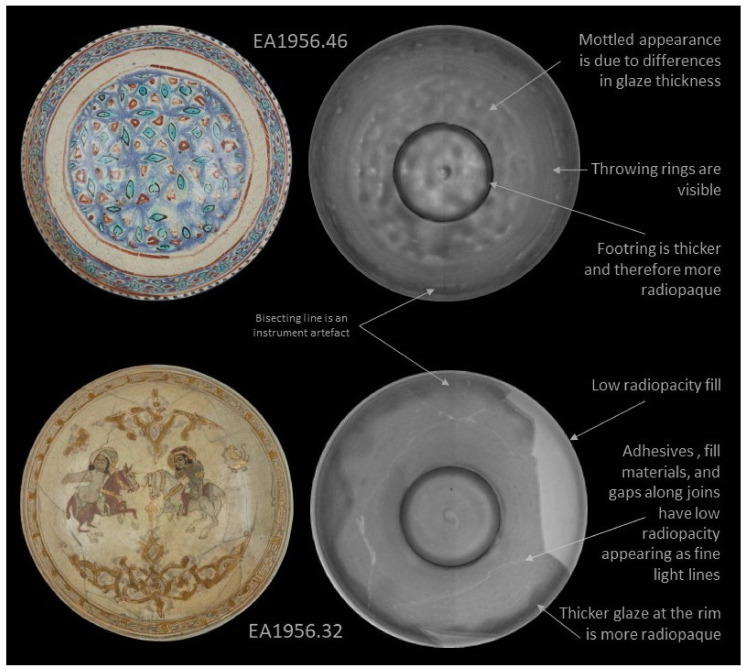
Results of digital photography on the left compared to digital radiographs on the right for EA1956.46 and EA1956.32.

**Figure 5:**
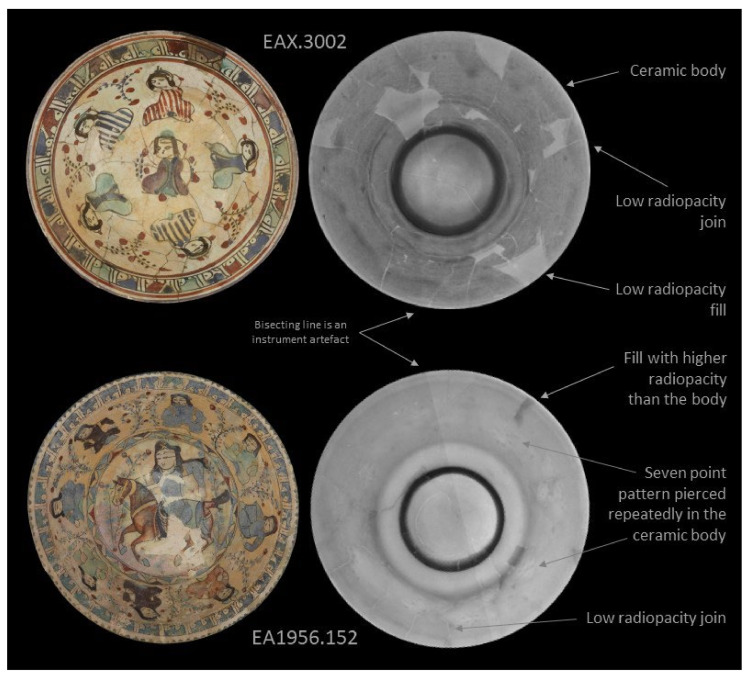
Results of digital photography on the left compared to digital radiographs on the right for EAX.3002 and EA1956.152.

**Figure 6:**
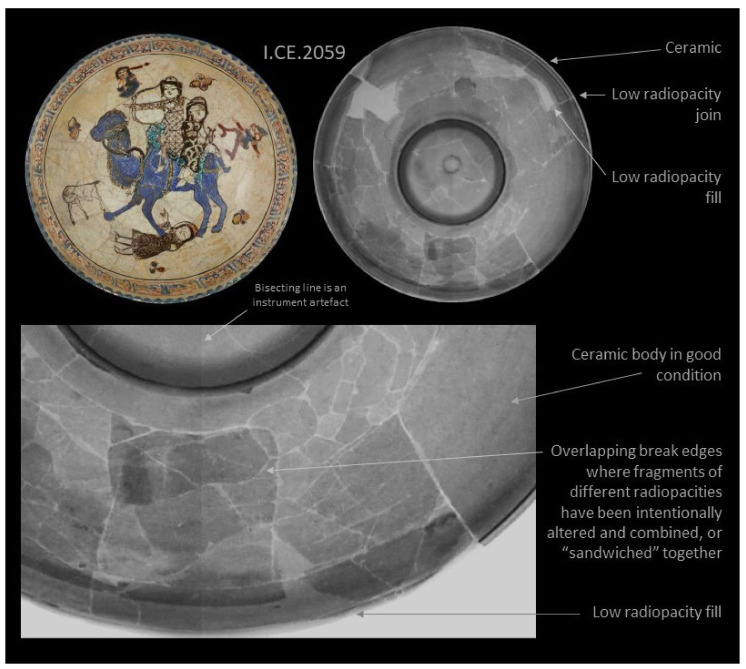
Results for I.CE. 2059, upper left digital photography, upper right digital radiograph, the detail below shows overlapping shards in the recumbent figure’s dress.

Radiography also yielded promising results, as seen in Figure 4, Figure 5 and Figure 6, where standard digital images and digital radiographs are presented. In these images lighter areas have lower radiopacity, and higher radiopacity areas are darker. Opacity in the radiographs is influenced both by thickness and composition. Note that it is possible to invert the grayscale of these images in the software. The radiography results are presented by complexity starting with EA1956.46 in Figure 4; this object is in pristine condition with no damage or restoration. The results show that original features in the bowl are visible, including throwing rings and differences in glaze thickness. The foot ring appears dark due to this area being much thicker and therefore, more radiopaque. It is also possible to see a spiral in the centre of the bowl, which is evidence of the potter’s hand. The radiograph of EA1956.32 is also presented in Figure 4; this bowl is broken into six large sections and has a large fill on the right side. The result for EA1956.32 is typical for a restored ceramic: there are fine light lines in the image where there are joins, darker areas at the foot ring and where the rim has a thicker application of glaze, and a large light area at the rim which is likely a lower density plaster fill.

The two bowls in Figure 5 have more complex conditions because both are broken into approximately two dozen shards with several smaller losses. The radiograph for EAX.3002 is as expected for a reassembled object, there are many fine light lines representing the joins, and the fills are less radiopaque than the ceramic, appearing lighter. The result for EA1956.152 did not follow this trend, even though the object was imaged under the same conditions as the rest of the sample set and did not have an obvious difference in the thickness of the vessel. In this radiograph, the ceramic body appears much lighter because it is less radiopaque than the other objects in this group. Elemental analysis using HH-XRF (handheld X-ray fluorescence) confirmed that the elemental composition of this object differed significantly from the other objects in this sample set. It is so light that the bisecting line, which is an instrument artefact, is much more prominent and could be misinterpreted as a feature of the bowl. In this image, the fills appear dark because they are more radiopaque than the ceramic body. It is also possible to see a seven-point pierced pattern which repeats around the vessel wall; see the annotations in Figure 5. The holes in this pattern are filled with glaze. Oddly, the polychrome overglaze enamel motif of seated figures and foliage has been painted over the pierced decoration in a way that does not integrate the two types of decoration in the overall design.

The result for I.CE. 2059 in Figure 6 is more complex, as there is an intricate network of joins, variation in the radiopacity of the ceramic shards, and overlapping break edges representing fragments which have been intentionally altered [10]. Two layers of ceramic are visible in parts of the bowl; this shows that ceramic fragments have been cut horizontally through the body in parallel to the glazed surface. Splitting shards allows motifs from the front and back to be matched independently, and the appropriate thickness achieved by “sandwiching” a layer of plaster or other fill material. An example of sandwiched shards can be seen in the dress of the figure beneath the camel. The technique is concisely illustrated by Norman’s case study of Dish LNS 309 C in the Victoria and Albert Museum [16]. It was understood from examination that this object was reassembled from small shards, but aspects of the condition were unclear before radiography.

## 4. Discussion

All three imaging techniques were found to be useful in the study of Mina’i ceramics because they supported and, in many cases, added information to visual examination. UV light is useful not only in highlighting areas of restoration, but also in indicating what the restorations are made of because the colour of fluorescence can indicate the type of material [14]. It is important to understand that not all restorations fluoresce, that some materials fluoresce more as they age, and that some unscrupulous restorers select their materials based on their appearance under UV light [6]. Although UV examination is common and useful [8,9,17], images under UV are not taken regularly because a completely dark space, a more powerful UV lamp, a camera with a manual focus and shutter speed, and a competent photographer are required [14]. The results of this project have shown that the investment in space and equipment to take high resolution UV images is warranted for studying Mina’i ceramics.

IR and UV images complimented each other in this project because they highlighted different aspects of restoration. IR images make it possible to distinguish differences within blocks of colour because inorganic pigments in the restoration and colourants in the ceramic can respond differently under IR light [6,18]. Not all pigments and colourants behave this way, but cobalt blues, copper turquoises and iron reds in glazes often did in this project. Tonal differences in infrared images are subtle and require patience to identify, but once the image is understood, a great deal of information can be gleaned from it. 

Not all restoration materials are highlighted by UV and IR imaging techniques; this is common in black paint [6]. No significant change in the appearance of the black paint used in restorations on Mina’i objects in this study was observed in UV or IR images. The lack of response from the black paint systems used in restorations is problematic because inscriptions and outlines in the iconography are painted in this colour. We can clearly see that some of these decorative elements sit on top of fills, but the extent of the black retouching remains obscure in most of the images. 

Radiographs are extremely useful in understanding the structural condition of objects which have been heavily overpainted [6]. Joins, which are low density voids, and fills are clearly visible in these images because fill materials have a different radiopacity than the ceramic. Radiography of these artworks indicated that some have ceramic material from other objects incorporated into them, a phenomenon which is not unheard of in Islamic ceramics [16,19]. Exposing ceramic objects to X-rays can alter the results of thermoluminescence testing [6,8,20], a dating technique commonly used in the commercial art market. Whether or not the object will be TL tested should be considered before radiography. However, this technique requires a sample, and the result will only correspond to that section of ceramic [21], making it inappropriate for most Mina’i objects. 

## 5. Conclusions

The results of this project have demonstrated that combining digital imaging under visible and UV light, IR, and radiography is a powerful tool for the non-invasive study of Mina’i ceramics. Capturing these images as a package gives a comprehensive understanding of the object’s surface and structural condition. Digital images are easily shared, creating opportunities for international collaborative research on typology, and radiographs have been shown to be particularly useful for understanding the structural condition which conservators can use to allocate treatment time accurately. 

In the past, these techniques were often outside the reach of collectors and small laboratories, due to cost, and health and safety restraints [8,14,17]. However, many collections have already made the initial investment in digital photography equipment and space. Acquiring an appropriate UV lamp is a sound investment because the technique is transferable across collections regardless of material. IR, which is more often associated with paintings, drawings, and textiles [6,18], has been shown to have significant application in Mina’i ceramics, and is the least complex to set up because there is no need for special safety controls or a dedicated space. The cost of radiology has come down significantly, and portable equipment can be hired for projects from companies such as SCANNA [22]. 

With these conclusions in mind, the authors propose the following guidelines for consistency in the collaborative study of Mina’i ceramics:

- Standard digital images should be taken on a black background with a measurement scale and colour card at the highest resolution reasonably practicable with the available equipment.
- Images under UV light should be taken after each shot so that the position and focus match the visible light image. Appropriate PPE should be worn.
- IR images should be taken in the same position on a black background, either during the same session as visible and UV, or by using those images as a reference.
- The position for digital radiographs should match the other images, TL testing should be ruled out before radiography, and a risk assessment done for safety.
- Notes and annotated images should be made before imaging, during UV examination, and after IR and radiography.

In future experimenting with other imaging techniques, parameters and filters might add to this package. Promising techniques include hyperspectral imaging, reflectance transformation imaging (RTI), computed tomography scanning (CT), and X-ray fluorescence (XRF) mapping. Results from these and other types of imaging of Mina’i ceramics would be interesting for comparison with this project.

## Data Availability

Not applicable.
